# Inflammatory mechanisms of Ginkgo Biloba extract in improving memory functions through lncRNA‐COX2/NF‐κB pathway in mice with status epilepticus

**DOI:** 10.1111/cns.14019

**Published:** 2022-11-23

**Authors:** Xiaopei Zou, Si Liu, Huihui Zou, Wanfei Zhou, Huaili Fu, Jiana Wei, Jiakang Zhang, Haoxuan Zeng, Tian Tan, Wenbin Zhou, Heyong Wu, Xinrun Chen, Xianju Zhou

**Affiliations:** ^1^ Special Medical Service Center, Neuroscience Center, Integrated Hospital of Traditional Chinese Medicine Southern Medical University Guangzhou China; ^2^ Cancer Center, Integrated Hospital of Traditional Chinese Medicine Southern Medical University Guangzhou China; ^3^ Department of Clinical medicine The First Clinical College of Guangzhou Medical University Guangzhou China

**Keywords:** Ginkgo biloba extract, LncRNA‐COX2, memory disorder, neuroinflammation, NF‐κB signal, status epilepticus

## Abstract

**Purpose:**

This study was to explore whether Ginkgo biloba extract (GBE) improve memory impairment by alleviating neuroinflammation signaling in mice with status epilepticus.

**Methods:**

The status epilepticus (SE) mice model was established by pilocarpine and treated with 100 mg / kg of GBE for 14 days. Spontaneous alternation of Y‐maze and new object recognition were used to explore memory impairment. To examine glial cell activation, we performed immunohistochemistry and immunofluorescence staining. The activation of NF‐κB signaling and the expression level of lncRNA‐COX2 were detected by Western blot and qRT‐PCR, respectively. Adeno‐associated virus lncRNA‐COX2 was injected into mice for overexpression of lncRNA‐COX2.

**Results:**

After GBE treatment, the spontaneous alternation rate and the recognition coefficient in SE mice were both increased. Moreover, activation of glial cells, NF‐κB signaling and lncRNA‐COX2 were significantly decreased in SE mice.

In the GBE‐treated SE mice with lncRNA‐COX2 overexpression, NF‐κB signaling was up‐regulated again; the reduced level of inflammation factors was reversed; the GBE‐rescued spontaneous alternation rate of Y‐maze was eliminated.

**Conclusion:**

Our results suggested that GBE reduces the hippocampal inflammation by down‐regulating lncRNA‐COX2 / NF‐κB signaling in the SE mice, leading to the decrease of neuronal damage and the improvement of memory functions.

## Graphical abstract

This study suggested that GBE reduces the hippocampal inflammation by down‐regulating lncRNA‐COX2/NF‐κB signaling in the SE mice, leading to the improvement of memory functions.

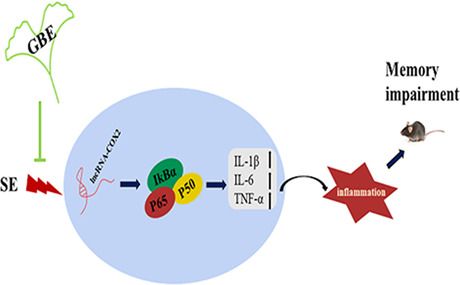

## 1 INTRODUCTION

Status epilepticus (SE) is a clinical neurological emergency with a high morbidity and mortality rate.^1^ SE‐induced hippocampal sclerosis, marked by loss of pyramidal neurons and proliferation of glial cells, eventually results in cognitive impairment.^2^ While benzodiazepines are the first‐line therapies for SE, 40% of patients still develop to refractory SE.^3^ Moreover, there is evidence that the refractory SE patients may have the neuroinflammation etiology similar to marginal encephalitis, as a result, immunotherapy plays a certain role.^4^

It is generally believed that acute inflammation occurs after SE, including brain inflammation and systemic inflammation. Levels of proinflammatory cytokines, such as IL‐6, IL‐8 and CXCL10, were observed elevation in cerebrospinal fluid of SE patients.^5^ SE patients showed increased neutrophil‐lymphocyte ratio and decreased albumin level.^6^ Transcriptome sequencing analysis from the SE rat hippocampus revealed that three of the five hub genes were inflammatory mediators: Tlr2, Stat3, and Ptgs2.^7^ Moreover, inflammation, as a potential trigger of seizure, aggravates seizure in frequency and duration. Sequencing data from temporal lobe epilepsy (TLE) patients suggested a positive correlation between Tlr2, Stat3 and seizure frequency.^7^ Excessive high mobility group box 1(HMGB1), a rich nuclear and cytoplasmic protein in mammalian cells, was released after cell death or through active secretion, further resulted in inflammation.^8^ These results suggested that HMGB1 may trigger seizures by inflammation pathways.^9^ Lipopolysaccharide (LPS) is an effective inducer of inflammatory signaling, exacerbating inflammatory factors in peripheral and central systems of mice.^10^ A noticeable increase in the severity of epileptic seizures and a decrease in the latency were observed in pilocarpine‐induced epilepsy model mice with LPS pretreatment.^11^ Medicinal plants with anti‐inflammatory activities can significantly lower the latency and duration of epileptic seizures, supporting the role of inflammation in epileptogenesis.^12^

It is well known that plenty of neurons are lost after SE. The autopsy results of five patients died of SE confirmed the density of hippocampal pyramidal neurons of these patients decreased significantly. Also, MRI studies indicated that the hippocampus of SE patients had progressive atrophy.^13^ Following studies suggested that inflammation may play a significant role in these processes. Rosiglitazone, a peroxisome proliferator‐activated receptor γ (PPAR γ) agonist, has been proved to inhibit microglia activation in the SE mice and rescued the damage of hippocampal neurons.^14^ Furthermore, it is reported that transient receptor potential vanillin 4 (TRPV4) activation enhanced inflammation and promotes the release of proinflammatory cytokines in various tissues and cells.^15^ Similarly, activation of TRPV4 enhanced neuroinflammation in the SE mice, while TRPV4 antagonist treatment significantly increases neuronal survival.^16^ Thus, inhibition of neuroinflammation becomes a new strategy for epilepsy.

Epilepsy can eventually lead to severe cognitive impairment, especially in memory.^17^ Recent studies indicated inflammation also has a profound impact on cognitive impairment. Exosomes with potent anti‐inflammatory effects improve the performance of SE mice in object localization test and new object recognition test.^18^ LPS directly induces spatial learning and memory decline in mice, associated with the reduction of synaptic protein levels.^19^ Meanwhile, overactivation of microglia is associated with memory impairment.^20^ These findings indicate that epilepsy may lead to memory impairment by neuroinflammation. Thus, blocking neuroinflammation might alleviate epilepsy‐related memory impairment.

LncRNAs, defined as non‐protein‐coding RNAs longer than 200 nucleotide, play a broad role in a large number of cells through interaction with DNA, RNA or protein.^21^ Recent studies have shown lncRNAs play a non‐negligible part in neuronal apoptosis, neuroinflammation and oxidative stress.^22^, ^23^, ^24^ Significant elevation of inflammatory associated proteins such as COX2 and NF‐κB as well as activation of astrocytes were observed in brain tissues from TLE patients.^25^ It is noteworthy that COX2/PGE2 and NF‐κB are increasingly recognized as vital participators in the SE inflammation.^26^, ^27^ Actually, the role of some lncRNAs in neuroinflammation is mostly related to NF‐κB signaling and NF‐κB related pathways.^28^, ^29^ For instance, lncRNA‐COX2 (also known as Ptgs2os2) acts as enhancer to promote the expression of Ptgs2 (a gene encoding COX2). Ptgs2 levels in lncRNA‐COX2 KO mice decreased significantly.^30^ lncRNA‐COX2 also showed a regulatory effect on the NF‐κB pathway, promoting the nuclear translocation and transcription of NF‐κB p65, and regulating the degradation of Ikb.^31^, ^32^ Delivery of lncRNA‐COX2 siRNA into the brain reduced the proliferation of microglia in mice.^33^ Similarly, knockdown of lncRNA‐COX2 decrease the number of activated microglia in animal models of multiple sclerosis.^34^ Therefore, we speculated that inhibition of lncRNA‐COX2 has the potential to inhibit SE‐related inflammation.

The use of ginkgo leaves can be traced back to the “Compendium of Materia Medica”. Because of its high medicinal value, ginkgo leaves are used as dietary supplements worldwide.^35^ Ginkgo biloba extract (GBE) is the concentrated extract of Ginkgo biloba leaves. Recent studies have shown that it inhibited the activation of primary microglia in rat.^36^ GBE also has anti‐inflammatory effect in cerebral ischemia model by regulating TLR4/NF‐κB, TXNIP/NLRP3 signaling axis, supported by a large number of findings.^37^, ^38^ Clinical studies in 2021 indicated GBE could improve cognition significantly in AD patients, even comparable to donepezil applied to AD treatment widely.^39^ Another clinical trial suggested that GBE could alleviate cognitive deficits after acute ischemic stroke.^40^ And some preclinical studies suggested that these benefits were probably owing to the anti‐inflammatory and antioxidant properties of GBE.^41^, ^42^, ^43^ However, little is known about whether GBE can improve cognitive functions by inhibiting neuroinflammation and its internal mechanisms in the SE mice. In view of the anti‐inflammatory effects of GBE in many neurological diseases and as a dietary supplement for Alzheimer's disease, this study was to explore whether GBE inhibited neuroinflammation by blocking lncRNA‐COX2/NF‐κB axis in the SE mice, eventually reducing neuronal loss and memory impairment.

## 2 RESULTS

### 2.1 GBE alleviates memory impairment induced by SE

**FIGURE 1:**
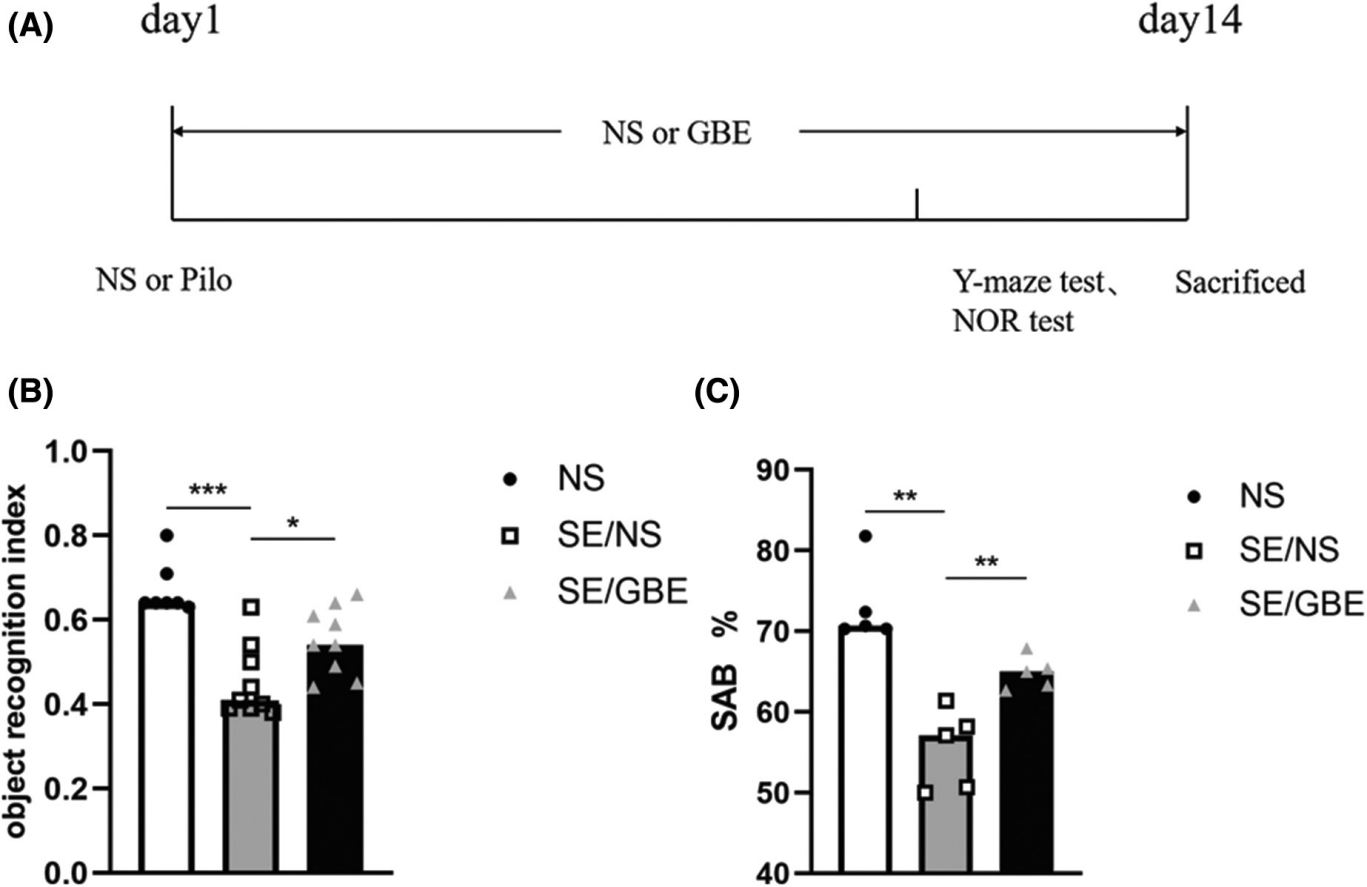
GBE alleviates memory impairment induced by SE.

Our results showed that the new object recognition index of the SE/NS group was lower than the NS group (Figure 1B, SE/NS group vs. NS group, 0.453 vs. 0.671, *p* < 0.001). Moreover, the index of the SE/GBE group was increased compared with that of SE/NS group (Figure 1B, SE/GBE group vs. SE/NS group, 0.551 vs. 0.453, *p* = 0.020). Full unedited blots are in Appendix S1.

In comparison with NS group, the SE/NS group showed a significant decrease in spontaneous alternation rate in Y‐maze (Figure 1C, SE/NS group vs. NS group, 52.83% vs. 73.87%, *p* = 0.008). The rate of SE/GBE group was higher than SE/NS group (Figure 1C, SE/GBE group vs. SE/NS group, 64.74% vs. 52.83%, *p* = 0.008). These results suggested that GBE alleviates memory impairment induced by SE.

### 2.2 GBE decreases neuronal loss in the SE mice

**FIGURE 2:**
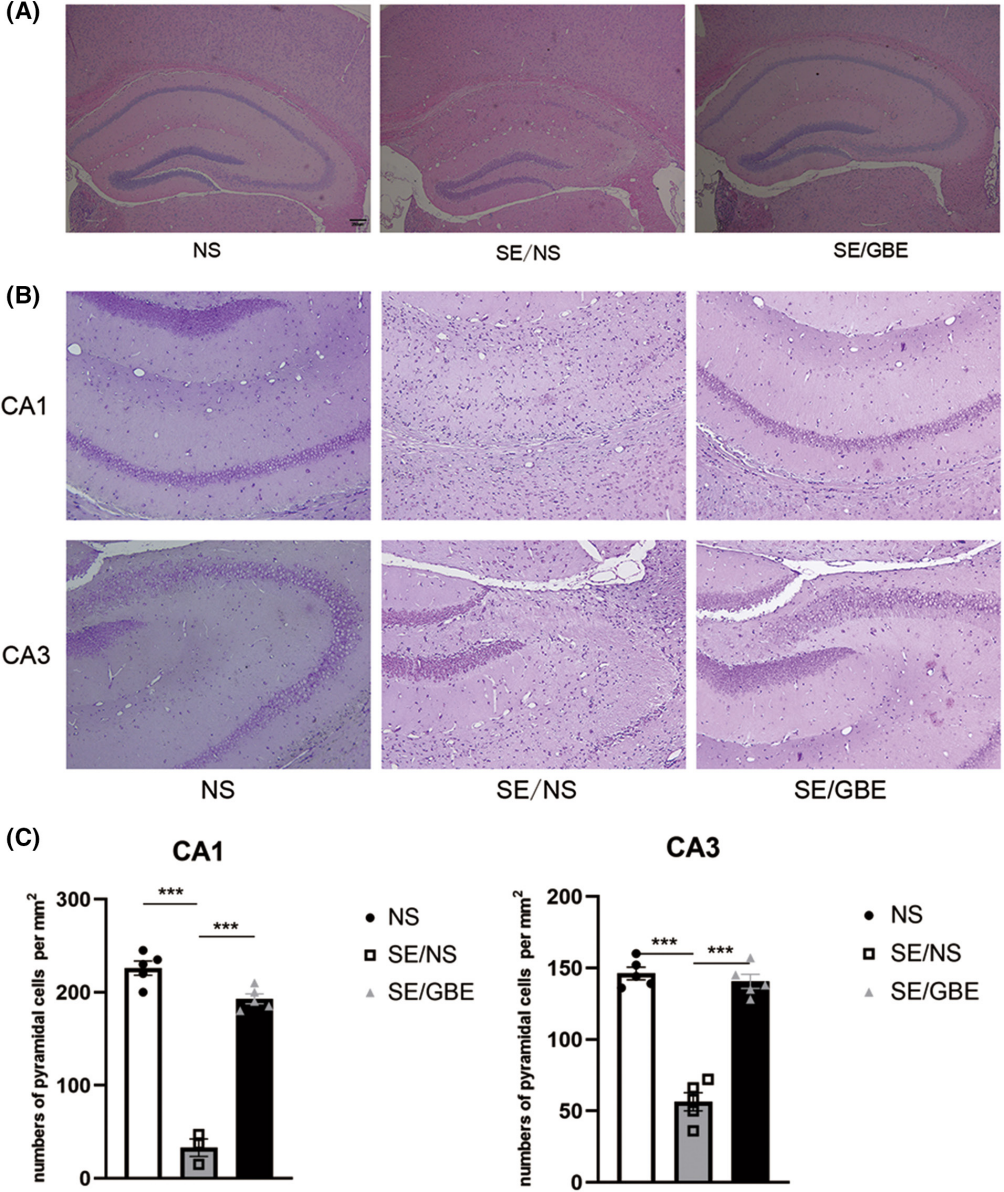
GBE decreases neuronal loss in the SE mice.

As shown in results of HE staining (Figure 2A), the hippocampal neurons in the NS group were arranged closely and regularly, and few neurons were lost. Neurons in the hippocampus of the SE/NS group were loosely arranged, irregularly shaped and seriously lost. The neurons in the SE/GBE group were less lost, although some cells were still irregular. In the NS group, Nissl staining revealed neat and compact arrangements of hippocampal neurons, while those in the SE/NS group were disordered and lost in large quantities (Figure 2B). The statistical results presented that neuronal number in CA1 (*p* < 0.001) and CA3(*p* < 0.001) of SE/NS group were much less than NS group(Figure 2C). And the number of neurons in CA1(*p* < 0.001) and CA3(*p* < 0.001) of SE/GBE group increased markedly comparing to the SE/NS group (Figure 2C).

### 2.3 GBE decreases neuroinflammation in the SE mice

**FIGURE 3:**
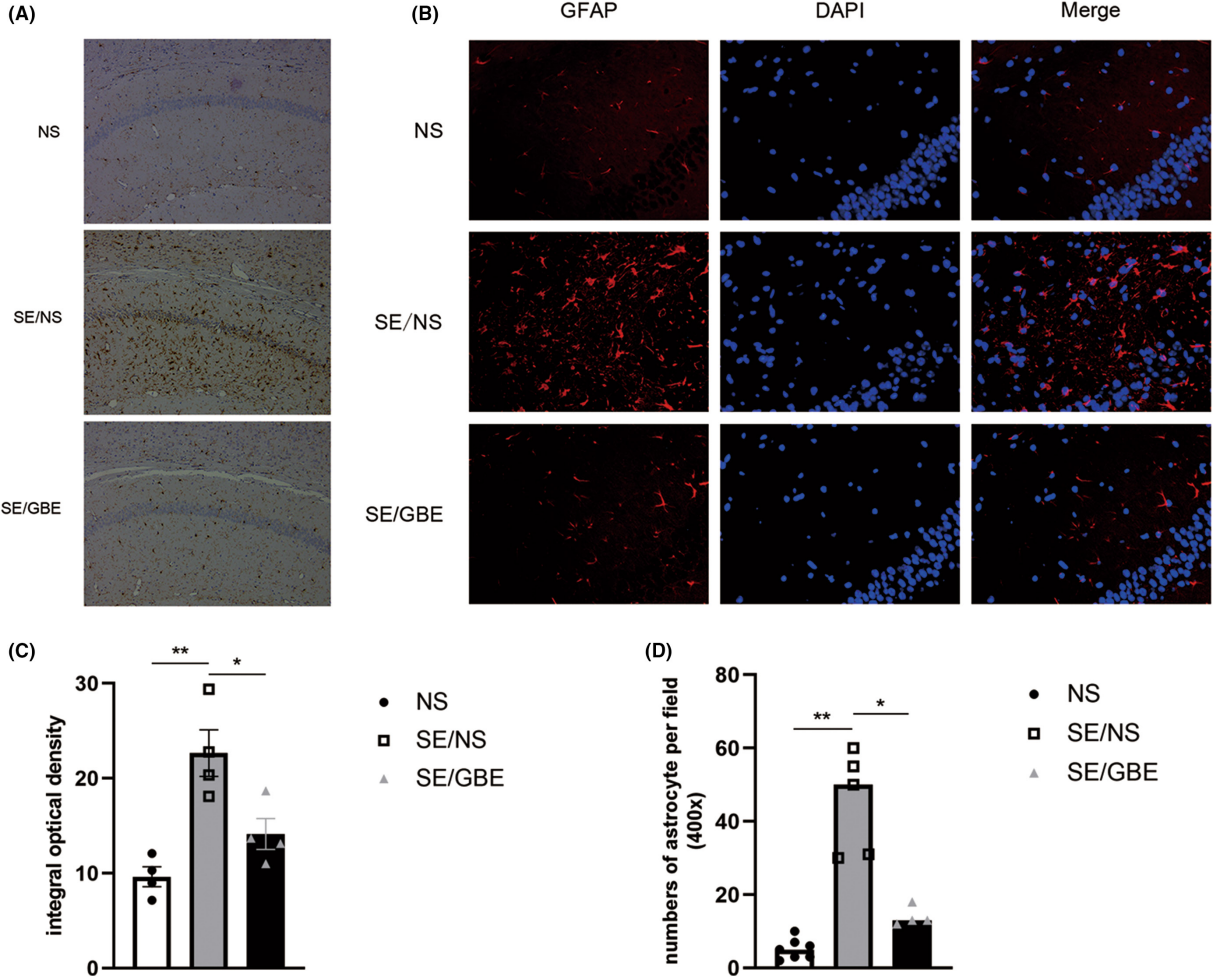
GBE decreases neuroinflammation in the SE mice.

In this study, the integral optical density of microglia showed a sharp increase in the SE/NS group than the NS group (*p* = 0.003). After GBE treatment, we observed that the density of the SE/GBE group was reduced compared to the SE/NS group (*p* = 0.027). Moreover, we found that microglia in the SE/NS group showed different morphology from the other two groups (Figure 3A,C), suggesting that GBE treatment can alleviate the inflammatory response after SE.

Our data revealed the number of astrocytes was increased in the SE/NS group than the NS group (Figure 3B,D) (*p* = 0.003). And a great decrease of the number was observed in the SE/GBE group (*p* = 0.016). Similarly, this evidence suggested that GBE treatment has a mitigation effect on hippocampal inflammation in the SE mice.

### 2.4 GBE inhibits lncRNA‐COX2/NF‐κB signaling pathways in the SE mice

**FIGURE 4:**
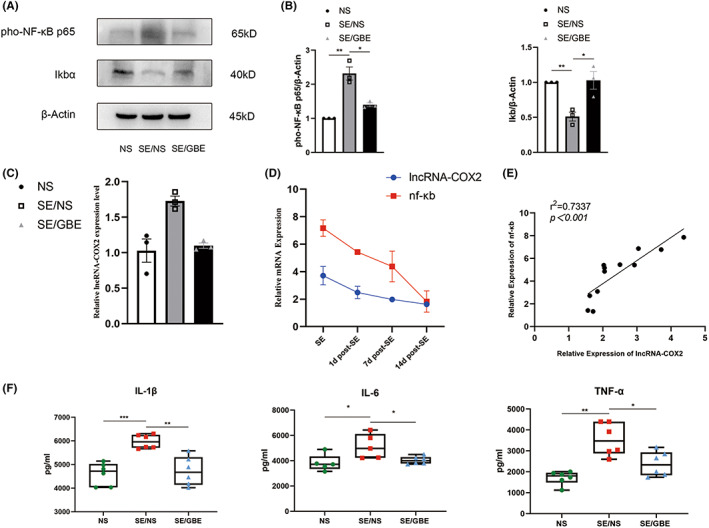
GBE inhibits lncRNA‐COX2/NF‐κB signaling pathways in the SE mice.

We detected the protein expression of NF‐κB pathway. As shown in Figure 4A,B, pho‐P65 in the SE/NS group was markedly up‐regulated compared to the NS group (*p* = 0.002). In comparison with the SE/NS group, the expression of pho‐P65 in the SE/GBE group presented a decrease (*p* = 0.010). And there was a higher expression level of Ikbα in the SE/NS group than the NS group (*p* = 0.002). We found that the expression of Ikbα in the SE/GBE group was up‐regulated compared to the SE/NS group (*p* = 0.022).

We detected RNA expression level of lncRNA‐COX2(Figure 4C). The results showed the lncRNA‐COX2 expression level in the SE/NS group increased by 1.6 times compared to the NS group (*p* = 0.018). At the same time, we found that the SE/GBE group showed a decline to 1.2 times relative to the NS group (*p* = 0.001). Conclusively, our results demonstrated that GBE inhibited up‐regulation of lncRNA‐COX2 in SE mice.

Studies have shown that lncRNA‐COX2 can activate NF‐κB pathway.^32^ Therefore, we speculated whether there was a correlation between lncRNA‐COX2 and NF‐κB. We found the RNA level of lncRNA‐COX2 and NF‐κB showed a similar decline trend over time, at SE and 1, 7 and 14 days after SE. (Figure 4D). As shown in Figure 4E, the correlation analysis revealed a strong positive correlation between them (*r* ^2^ = 0.734, *p* < 0.001). This evidence suggested that the expression of lncRNA‐COX2 and NF‐κB in SE mice was positively correlated.

As shown in Figure 4F, the IL‐1β, IL‐6 and TNF‐α levels in the SE/NS group presented remarkable increase compared to the NS group (*p* < 0.001, *p* = 0.036, *p* < 0.001, respectively). Compared to the SE/NS group, the levels of IL‐1β, IL‐6 and TNF‐α in the SE/GBE group were decreased by 21%, 21.4% and 33%, respectively(*p* = 0.002, *p* = 0.02, *p* = 0.015). To sum up, these results showed that GBE inhibited hippocampal inflammation after SE.

### 2.5 Overexpression of lncRNA‐COX2 reverses anti‐inflammatory effect and memory improvement of GBE in the SE mice

**FIGURE 5:**
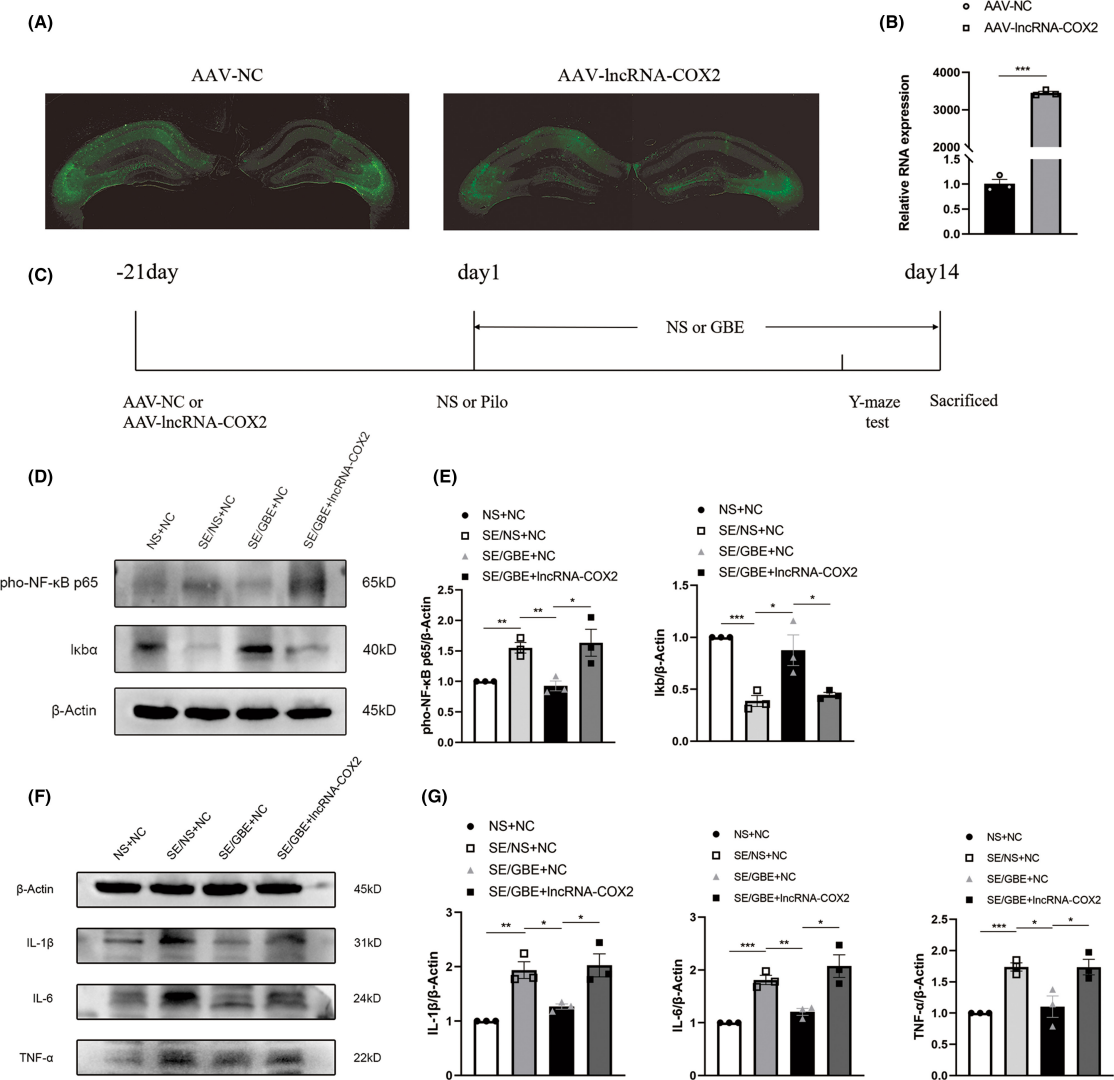
Overexpression of lncRNA‐COX2 reverses anti‐inflammatory effect of GBE in the SE mice.

Owing to the strong correlation between lncRNA‐COX2 and NF‐κB after SE, we speculated inhibiting lncRNA‐COX2 can account for the inhibitory effect of GBE on NF‐κB signaling. Therefore, to further explore the mechanism, we injected AAV‐GFP to overexpress lncRNA‐COX2 into the bilateral hippocampus of mice. Three weeks later, we confirmed the expression of lncRNA‐COX2 mediated by AAV, because GFP fluorescence specifically indicated the expression of vector in the hippocampus (Figure 5A). The qPCR results also showed higher expression of lncRNA‐COX2 in the mice injected with AAV‐lncRNA‐COX2 compared to the mice injected with AAV‐NC (Figure 5B, *p* < 0.001).

Compared to the SE/GBE + NC group, the SE/GBE + lncRNA‐COX2 group showed a higher expression level of p‐P65(Figure 5D,E) (*p* = 0.04). Then, we examined the protein level of proinflammatory factors in the lncRNA‐COX2‐AAV group. As shown in Figure 5F,G, in the SE/GBE + lncRNA‐COX2 group, the level of IL‐1β, IL‐6 and TNF‐α was up‐regulated compared with the SE/GBE + NC group (*p* = 0.025, *p* = 0.018, *p* = 0.040, respectively). These results suggested that overexpression of lncRNA‐COX2 can reverse inhibition of GBE on NF‐κB signaling pathway and inflammation in the hippocampus.

**FIGURE 6:**
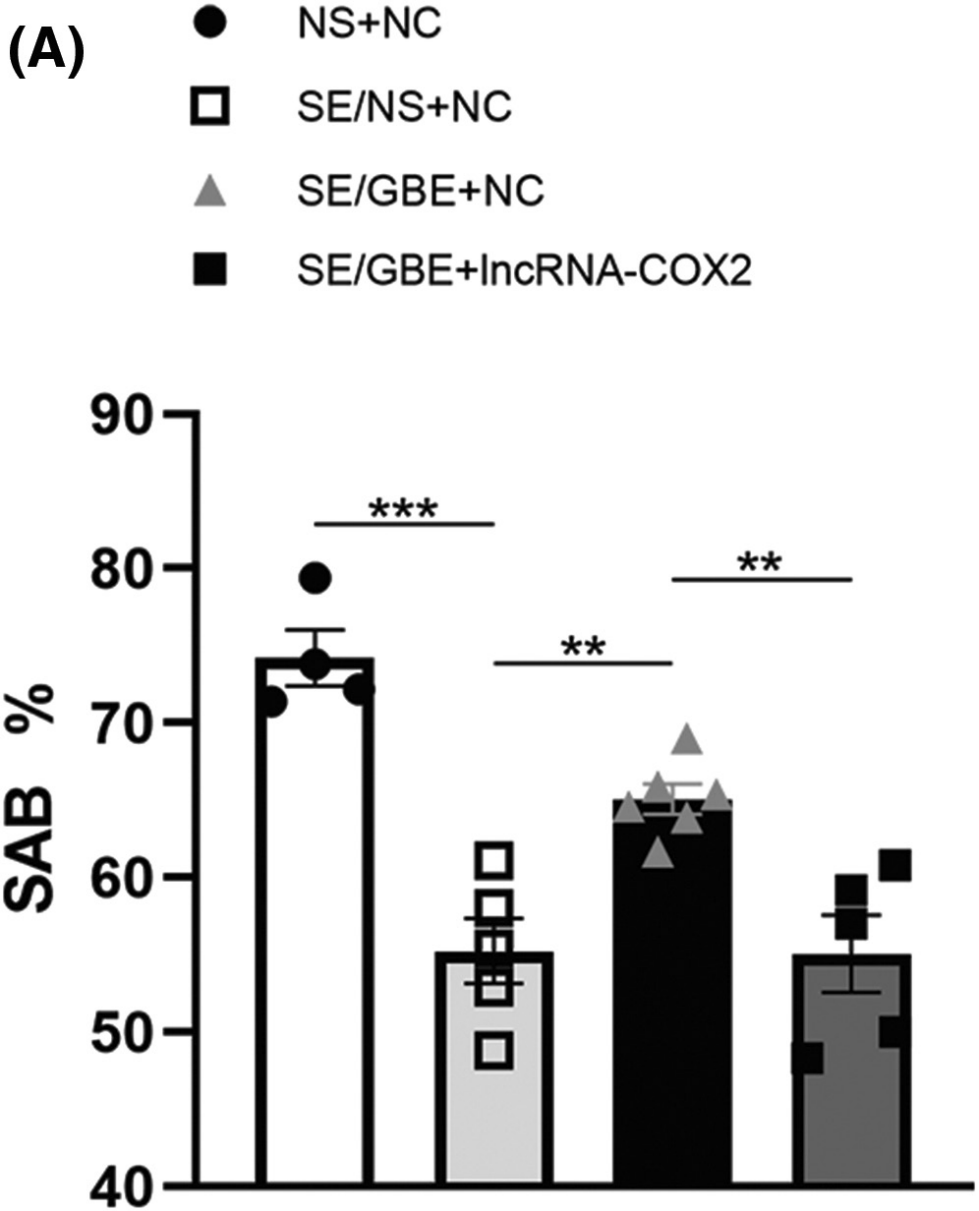
Overexpression of lncRNA‐COX2 reverses memory improvement of GBE in the SE mice.

In Y‐maze spontaneous alternation test, our outcomes implied that the spontaneous alternation rate of the SE/GBE + lncRNA‐COX2 group was decreased compared to the SE/GBE + NC group (Figure 6A, 55.06% vs. 65.08%, *p* = 0.004), suggesting that overexpression of lncRNA‐COX2 mitigated the effects of GBE on memory impairment after SE.

**FIGURE 7:**
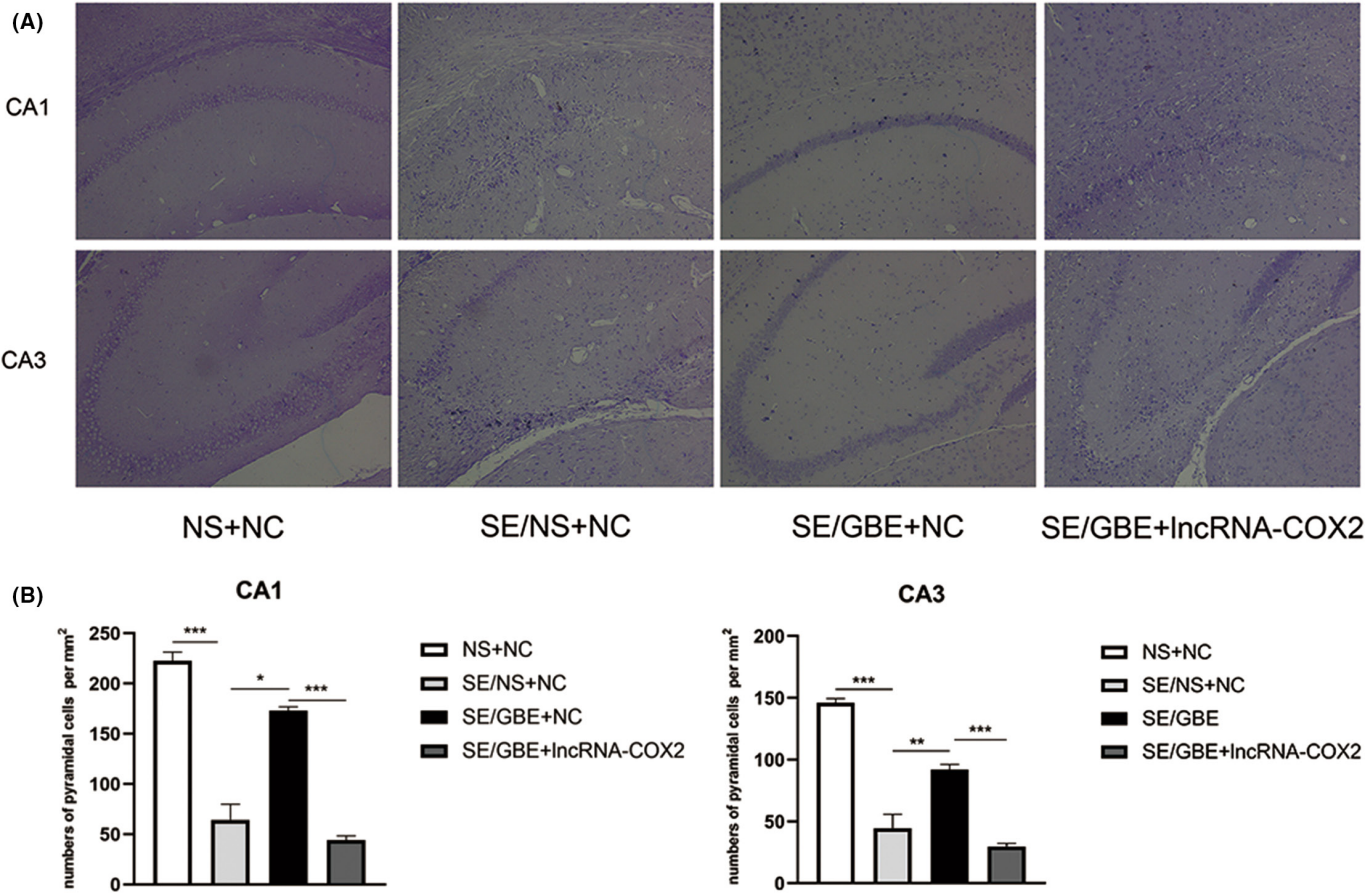
Overexpression of lncRNA‐COX2 reverses improvement of GBE on neuronal loss in the SE mice.

As shown in Figure 7A, our results of Nissl staining demonstrated that hippocampal neurons in the SE/GBE + lncRNA‐COX2 group were disordered and lost in large quantities. The statistical results presented that neuronal number in CA1 and CA3 of SE/GBE + lncRNA‐COX2 group were much less than the SE/GBE + NC group (Figure 7B, *p* < 0.001, *p* < 0.001).

## 3 MATERIALS AND METHODS

### 3.1 Experimental design

On day 1, SE mice were injected with GBE (the SE/GBE group) or saline (the SE/NS group) for 14 consecutive days to evaluate the effects of GBE treatment on memory impairment, neuron loss, and neuroinflammation. The NS group was only injected saline during SE establishment and during the subsequent treatment. The Y‐maze spontaneous alternation test or NOR test was carried out during the 14 days of continuous treatment. On day 14, then, mice were sacrificed for Western blot, immunohistochemistry or ELISA experiments (Figure 1A).

On day‐21, before SE establishment, AAV‐lncRNA‐COX2 or AAV‐NC were randomly injected into mice. On day 1 of SE establishment, the mice SE were given GBE (the SE/GBE + NC group, the SE/GBE + lncRNA‐COX2 group) or saline treatment (SE/NS + NC group) for 14 consecutive days to observe whether overexpression of lncRNA‐COX2 might reverse the therapeutic effect of GBE in SE mice. The NS + NC group was injected with AAV‐NC on the 21th day before SE establishment, and only saline was injected during the establishment of SE model and subsequent treatment. The Y‐maze spontaneous alternation test or NOR test was performed during the 14 days of continuous treatment. On day 14, mice were sacrificed for Western blot, or for immunohistochemistry experiments (Figure 5C).

### 3.2 Mice

Male C57BL/6 mice (20–22 g) from Beijing Yancheng Biological Company were raised for a few days access to sufficient water and food in a cycle of 12 h:12 h light‑dark before the experiment. All experiments were in line with the ethical requirements of the Animal Management and Use Committee of Southern Medical University. This study was approved by the Animal Management and Use Committee of Southern Medical University.

### 3.3 Establishment of SE model

To block the peripheral cholinergic side effects of pilocarpine, mice were injected intraperitoneally with scopolamine after weighing. Then pilocarpine was administered intraperitoneally to mice (300 mg/kg).The seizures of mice were observed, and the severity of seizures was determined by the modified Racine score.^44^ SE was defined as seizures ≥4 score, and lasted for 2 h. Two hours later, diazepam (7.5 mg/kg) was injected intraperitoneally to terminate seizures. Treatment with either 100 mg/kg GBE or saline was performed for 14 days in SE mice. Treatment dose was referring to previous articles.^43^, ^45^

### 3.4 Y‐maze spontaneous alternation test

Put the mice in the room to prepare for the test in advance. The mice were conducted spontaneous alternation test in a dark Y‐maze with 120° angle of each other, and the three arms were designated as A, B, and C, respectively. The mouse was placed from the distance of arm A, given free access to the maze for 8 min. Mouse with good spatial memory remembered the maze arms that it has visited and tended to enter the arms that had recently visited less. Continuous access to all three arms was the definition of alternating behavior. At the same time, it was recorded with a camera for subsequent analysis. Before the next mouse test, clean the maze with ethanol to avoid the feces and smell of the former mouse. Alternate percentage = alternate times/(total number of arm entries−2), high alternate percentage was regarded as good spatial memory.^46^

### 3.5 New object recognition test

New object recognition test needed to prepare a square box with walls around (40 cm× 40 cm). Mice were allowed to adapt to the test room before all tests began. The whole test was divided into three stages: the first stage: let mice adapt to the empty box without objects for 5 mins; the second stage: placed two identical objects symmetrically on one side of the box to keep the object from being knocked down by mice. Then gently put the mouse back to the two objects in the box, leaving the test room, free to explore for 10 mins; phase 3: Replaced one of these objects with new objects of similar size but different shapes. Then gently put the mouse back to the two objects in the box, leaving the test room, free to explore for 10 mins. Mouse activity was recorded by the camera above. Identification coefficient = new object exploration time/(new object exploration time + old object exploration time). The higher the recognition coefficient was, the better the recognition memory was.

### 3.6 HE staining

Paraffin sections (5 μm) were placed in 65°C oven for 1–2 h. Wash 3 min with pure water after hydration. Dye with hematoxylin for 5–6 min. Then, the slices were put into a dyeing tank filled with 1% hydrochloric acid and ethanol for about 5–10 s. Then dye with eosin solution for 2 min and rinse with water for 30 s. Finally, sliced into the following dyeing cylinder for dehydration and transparency: 75% ethanol 30 s, 85% ethanol 1 min, 95% ethanol 5 min, 100% ethanol 5 min, xylene I 10 min, xylene II 10 min. Slices are sealed with neutral resin.

### 3.7 Nissl staining

Paraffin sections were placed in 65°C oven for 1–2 h. The deparaffin step was the same as HE staining. Slices were stained with cresol purple at 37°C for 2 h. Then they were put into the differentiation solution for several seconds until the background was observed under the microscope to be nearly colorless and stopped. After neutral resin sealing, the neurons of the CA1 and CA3 regions were observed and photographed under the microscope, and the average neurons per mm^2^ were counted.

### 3.8 Enzyme‐linked immunosorbent assays

ELISA kits were purchased from the company (ABColnal). Used double antibody sandwich method according to kit instructions(IL‐1β, RK00006; IL‐6, RK00008; TNF‐α, RK00027). Preparation of standard: 1 ml standard diluent was added into standard freeze‐dried powder and diluted by concentration gradient. The steps were as follows: Incubation of samples for 2 h. Then, the biotin‐binding antibody was added and incubated for 1 h. Streptavidin‐HRP solution was added and incubated for 30 min. Then, the chromogenic substrate was added and incubated in dark for 20 min. Finally, the termination liquid was added. The sample concentration was calculated according to the measured OD value and standard curve.

### 3.9 Immunohistochemistry

Paraffin sections were placed in 65°C oven for 1–2 h. The deparaffin step was the same as HE staining. After washing slices with distilled water for three times, the slices were thermally repaired with antigen. After blocking endogenous peroxidase, the sheep serum was used for blocking. Then incubation overnight with primary antibodies at 4°C: anti‐rabbit antibody iba‐1(abcam, ab178847). Incubation with the secondary antibody for 1 h was followed by seal with neutral resin. The stained cells were examined and photographed under the microscope. Image J is used to calculate the average optical density of the image.

### 3.10 Immunofluorescence staining

The steps are basically the same as immunohistochemistry. Penetrated with 0.3% Triton‐100, blocked the slice for 1 h and incubated with primary antibody overnight: 4°C, anti‐rabbit antibody gfap (abcam, ab68428). Took care to avoid light when the second antibody was incubated, then dropped dapi solution for 10 min, and finally observed and took photos. The number of gfap^+^ in 400 × field of vision was calculated.

### 3.11 Quantitative real‐time PCR

The following experiments were performed using PrimeScript RT kit (Takara, RR047A). There were 20 μl of reaction system, with conditions of 37°C 15 min, 85°C 5 s. TB Green Premix Ex Taq (Takara) was used for quantitative real‐time PCR amplification through LightCycler 480 II system (Roche). The reaction conditions are: 95°C 30 s, 95°C 5 s, 60°C 20 s, a total of 40 cycles. Using GAPDH as a reference gene, the primers listed in Table 1 were those used in this experiment. Relative quantification was detected by 2^−ΔΔCt^ method. Three copies per experiment were used to obtain the average value.

**TABLE 1:** Primer sequences

GAPDH forward	ATGACTCTACCCACGGCAAG
GAPDH reverse	TACTCAGCACCAGCATCACC
lncRNA‐COX2 forward	TCCTTTCCCCCTCAATTCTT
lncRNA‐COX2 reverse	TTTTCCCAATCTGCTTTGGT
nf‐κb forward	TCGGGACAAACAGCCTCG
nf‐κb reverse	GTTCCTGGTCCTGTGTAGCC

### 3.12 Western blot

The hippocampus of mice was removed on ice and RIPA lysate was added. A total of 30 μg protein was added for sulfate‐polyacrylamide gel electrophoresis (SDS‐PAGE) and transferred to PVDF membrane wetly. After blocking for 1 h, incubation with primary antibody at 4°C overnight ((β‐actin, ABclonal) (phosph‐p65, Cell Signaling Technology) (ikb, Cell Signaling Technology) (IL‐1β, Proteintech) (TNF‐α, Proteintech) (IL‐6, Proteintech)). Incubated with second antibody for 1 h. Finally, visualize it using a chemiluminescent reagent (Millipore). Quantification of the levels of protein normalized to β‐actin.

### 3.13 Stereotaxis injection

AAV vector for overexpression of lncRNA‐COX2 were constructed and produced by OBiO Technology Corporation, Shanghai, China. Mice were anesthetized with chloral hydrate, followed by stereotaxic injection. AAV‐NC or AAV‐lncRNA‐COX2 with GFP fluorescence labeling was injected into the hippocampus (AP‐2.0 mm, LM ± 1.8 mm and DV‐2 mm) at a rate of 0.2 μl/min using a Hamilton injector. After injection, in order to prevent virus reflux, the syringe was kept in situ for 8 min and then removed. We detected the fluorescence of hippocampus at 1, 2 and 3 weeks after injection of AAV‐NC and AAV‐lncRNA‐COX2 because GFP fluorescence specifically indicated the expression of vector in the hippocampus. Finally, we found that at the third week, different from the first and second weeks, all parts of the hippocampus were fluorescent.

### 3.14 Statistical analysis

The data were analyzed with GraphPad Prism6 (San Diego, CA, USA). The measured data are expressed as mean ± SD. We used Levene's test to ensure the homogeneity of standard deviation, and performed Shapiro–Wilk test to ensure the normality. Mann Whitney test or Student *T*‐test was performed to compare the average values between the two groups. The Spearman *X* ^2^ test was used for the correlation analysis between lncRNA‐COX2 and NF‐κB. The Statistical significance was defined as *p* value <0.05.

## 4 DISCUSSION

Studies have found that active ingredients of GBE can alleviate memory impairment in epileptic rats.^47^ In our study, we reported that memory improvement of GBE in SE mice was related to its anti‐inflammatory effects. Further, we found that anti‐inflammatory effects of GBE in SE mice were mediated by lncRNA‐COX2/NF‐κB pathway.

Recent clinical studies have shown that GBE is as effective as donepezil in improving cognition of Alzheimer's patients.^39^ Cognitive deficits after acute ischemic stroke and hemorrhagic stroke can also be attenuated by GBE.^40^, ^48^ Moreover, GBE can reduce cognitive impairment caused by toxic substances such as trimethyltin and bisphenol A.^43^, ^49^ There are still few studies on GBE alleviating cognitive deficits in epilepsy. Our study provided evidence that GBE has a mitigating effect on memory impairment in the SE mice.

Mounting studies have shown GBE exerts anti‐inflammatory effects on both the cardiovascular system and the central nervous system. For instance, GBE can alleviate viral myocarditis by inhibiting matrix metalloproteinase‐3 (MMP‐3), a protein reported to mediate immune cell migration and cytokine secretion.^50^ The reduction of atherosclerosis by GBE in diabetic model mice is related to the effect of GBE on reducing inflammatory cytokines.^51^ It was reported that GBE can inhibit the inflammatory response to reduce early brain damage in rats with subarachnoid hemorrhage.^37^ GBE can also inhibit Aβ1‐42‐induced inflammatory responses in microglia.^52^ Consistently, our results showed that GBE attenuates SE‐induced neuroinflammation.

Previous studies suggested that GBE alleviates cognitive impairment in epileptic animals by increasing BDNF, neuropeptide Y or increasing the differentiation of neural stem cells. Our study showed that GBE improved memory functions in SE mice through reducing neuroinflammation, including NF‐κB inflammation pathway. Consistently, Ginkgolide B reduces neuroinflammation and alleviates learning and memory impairment in vascular dementia rats by regulating NF‐κB pathway^53^; Moreover, GBE improves cognitive function in elderly db/db^−/−^ diabetic mice by regulating beclin‐1 and NF‐κB signaling pathways^54^; Also, the therapeutic effect of ginkgolide B on perioperative neurocognitive dysfunction is related to its antioxidant effect.^55^ We further revealed the regulation role of lncRNA‐COX2 in the neuroinflammation of SE, which was consistent with previous studies.^34^ We suggested that lncRNA‐COX2 was located upstream of NF‐κB by overexpression f lncRNA‐COX2 leading to the reactivation of NF‐κB pathway, but we did not provide evidence on their interaction.

Previous studies showed that lncRNA‐COX2 promotes nuclear translocation of NF‐κB, and that lncRNA‐COX2 regulates Ikbα degradation.^32^, ^34^ As an enhancer of Ptgs2, lncRNA‐COX2 plays an essential role in the regulation of COX2. There is evidence that NF‐κB expression decreased in COX2 knockout mice.^56^, ^57^ Thus, there might be a crosstalk between COX2 and NF‐κB. The association between the expression of lncRNA‐COX2 and NF‐κB in our study also suggested the crosstalk. Additionally, the reversal results after overexpression of lncRNA‐COX2 might be related to COX2, which indirectly increased the level of NF‐κB. However, our study failed to explore whether GBE has a regulatory effect on COX2.

## 5 CONCLUSION

Our results showed that SE induces hippocampal inflammation, neuronal loss and memory impairment. GBE alleviated SE‐induced neuroinflammtion, neuronal loss and memory impairment in the mouse hippocampus by inhibiting lncRNA‐COX2/NF‐κB inflammation signaling. Therefore, GBE may be an effective strategy for memory impairment induced by epilepsy.

## AUTHOR CONTRIBUTIONS

X.P.Z. and S.L. designed the study, completed the experiment, collated the data, and contributed to modifying the manuscript. X.P.Z. produced the initial draft of the manuscript. X.J.Z. designed the study, provided research funds, and were responsible for the revision of the entire manuscript. H.H.Z., W.F. Z., H.L.F., J.N.W., J.K.Z., H.X.Z., T.T., W.B.Z., H.Y.W., and X.R.C. participated in the course of the experiment. All authors have read and approved the final submitted manuscript.

## FUNDING INFORMATION

This work was supported by Traditional Chinese Medicine Bureau of Guangdong Province. (Grant No.20203012).

## CONFLICT OF INTEREST

All coauthors had no conflicts of interest to disclose.

## Supporting information


AppendixS1

## Data Availability

The data that supports the findings of this study are available in the supplementary material of this article.
