# Supplementary material for: Inflammatory mechanisms of Ginkgo Biloba extract in improving memory functions through lncRNA‐COX2/NF‐κB pathway in mice with status epilepticus
Source: CNS Neurosci Ther. 2022 Nov 23;29(1):471–82. doi: 10.1111/cns.14019 (PMC9804085; doi:10.1111/cns.14019)

Full unedited blot for Figure4A pho-NF-κB p65

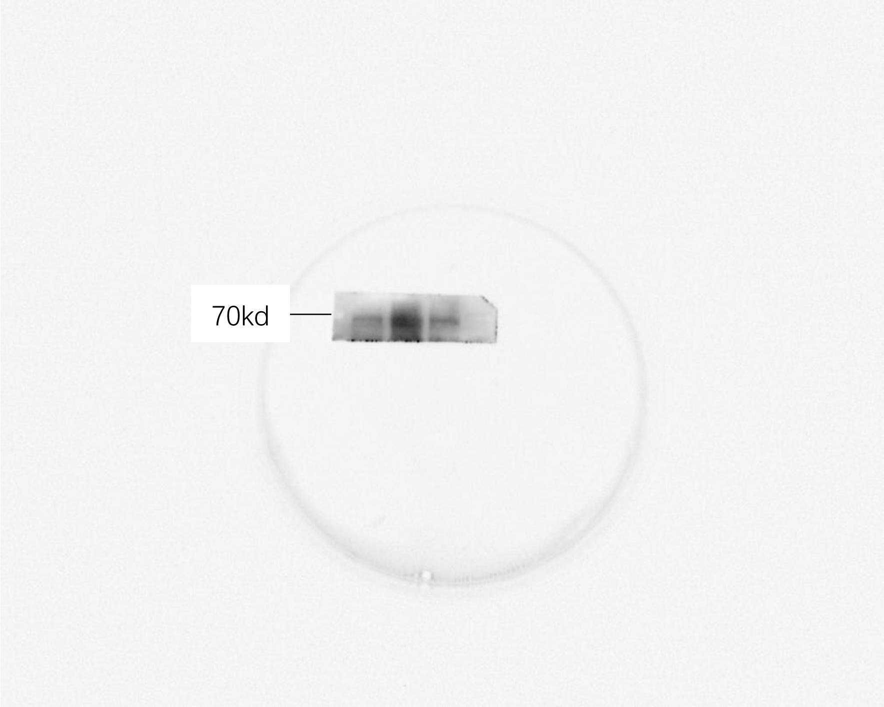

Full unedited blot for Figure4A Ikbα

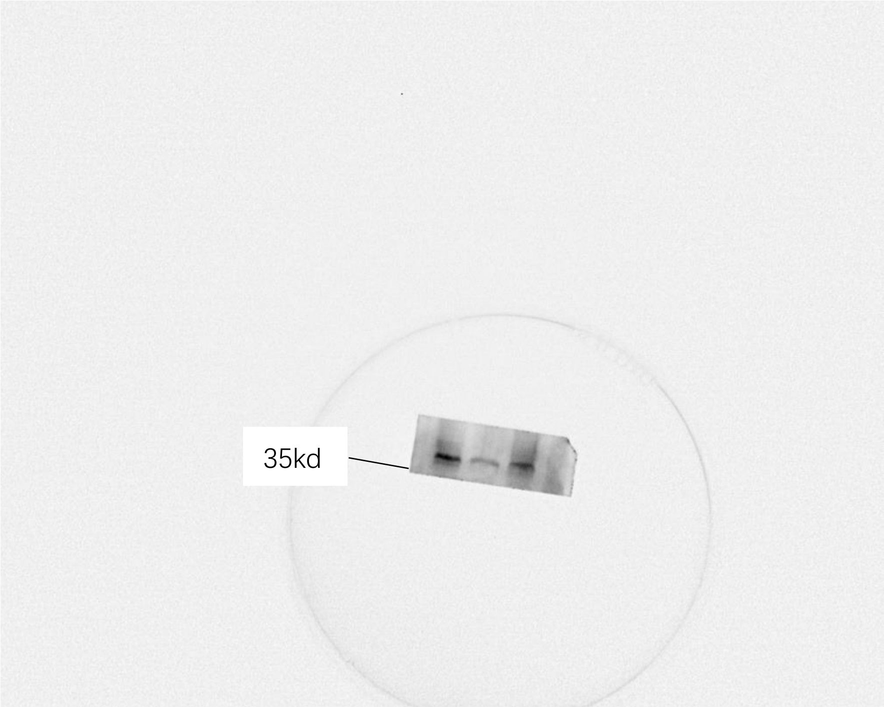

Full unedited blot for Figure4A β-Actin

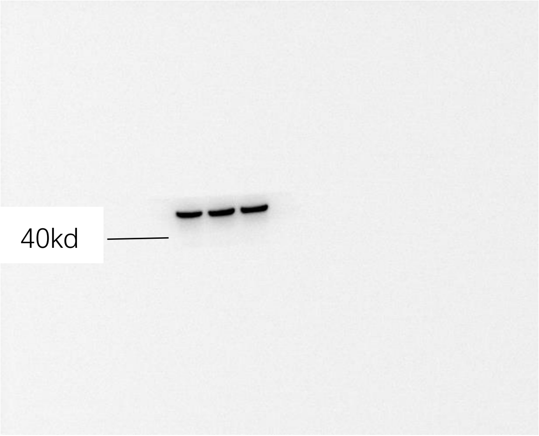

Full unedited blot for Figure5D pho-NF-κB p65

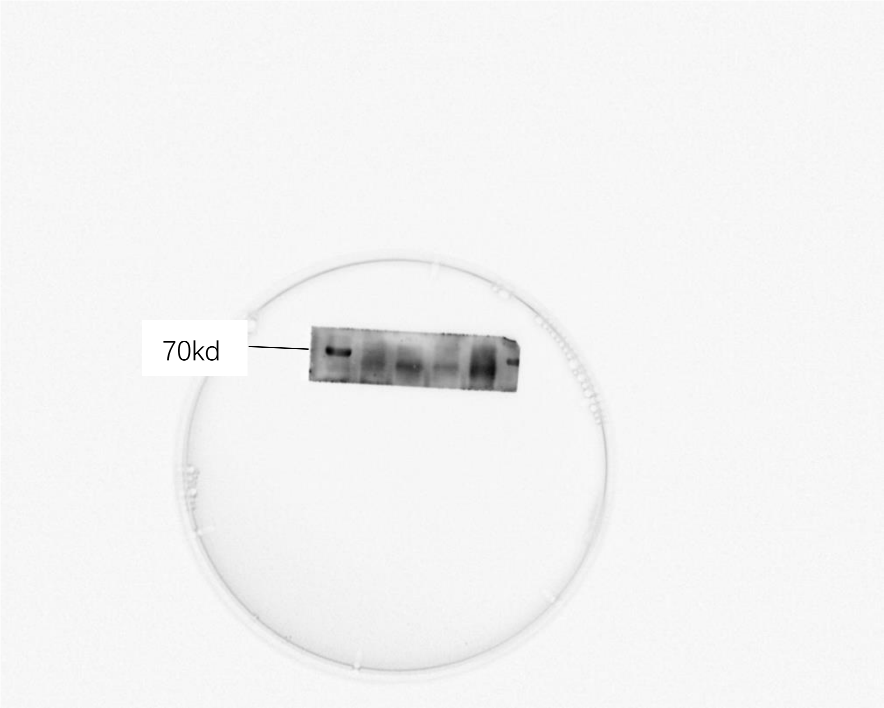

Full unedited blot for Figure5D Ikbα

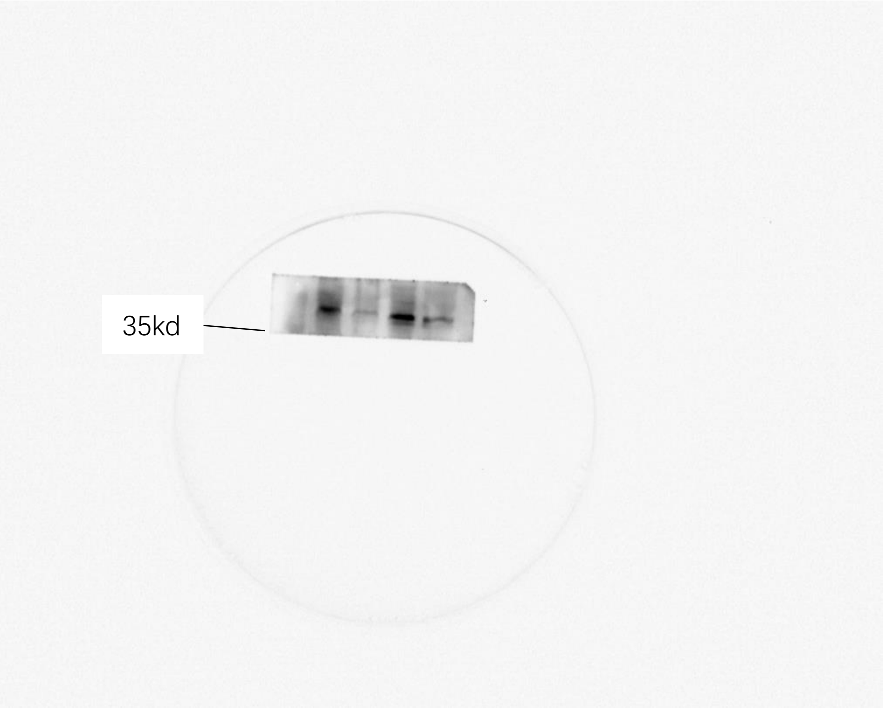

Full unedited blot for Figure5D β-Actin

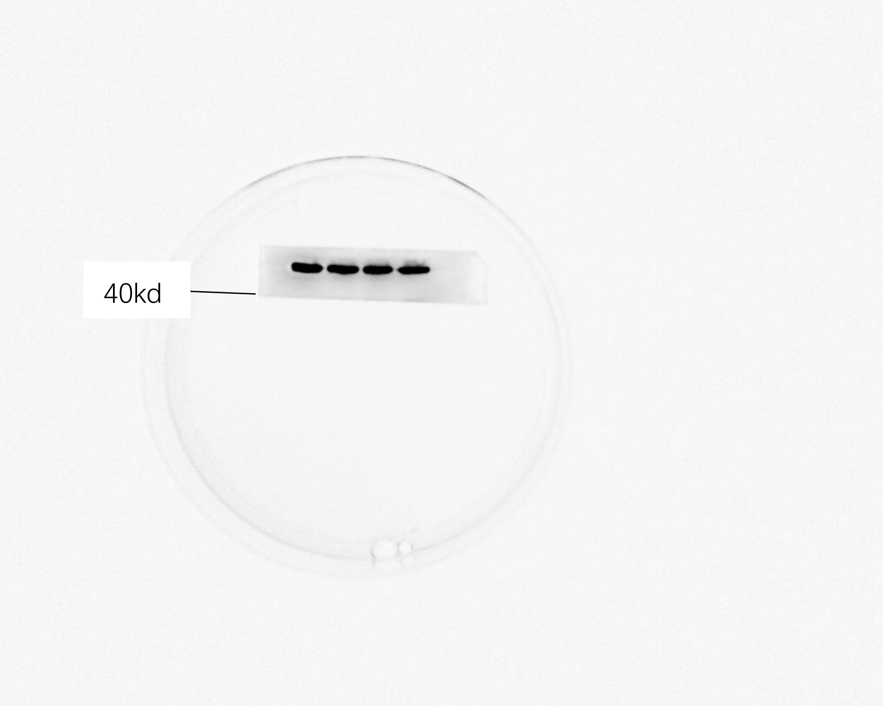

Full unedited blot for Figure5F β-Actin

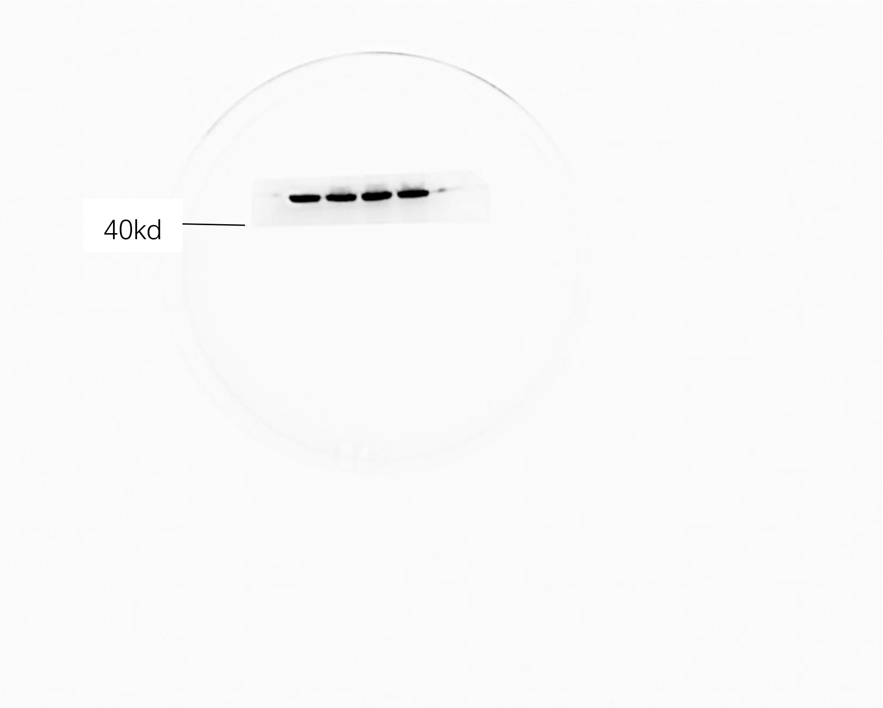

Full unedited blot for Figure5F IL-1β

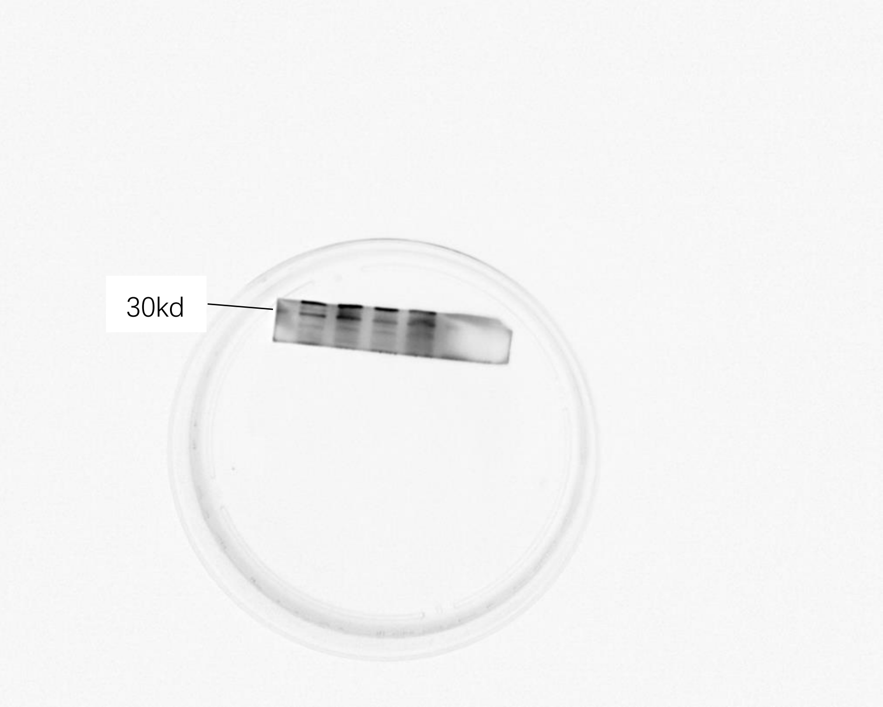

Full unedited blot for Figure5F IL-6

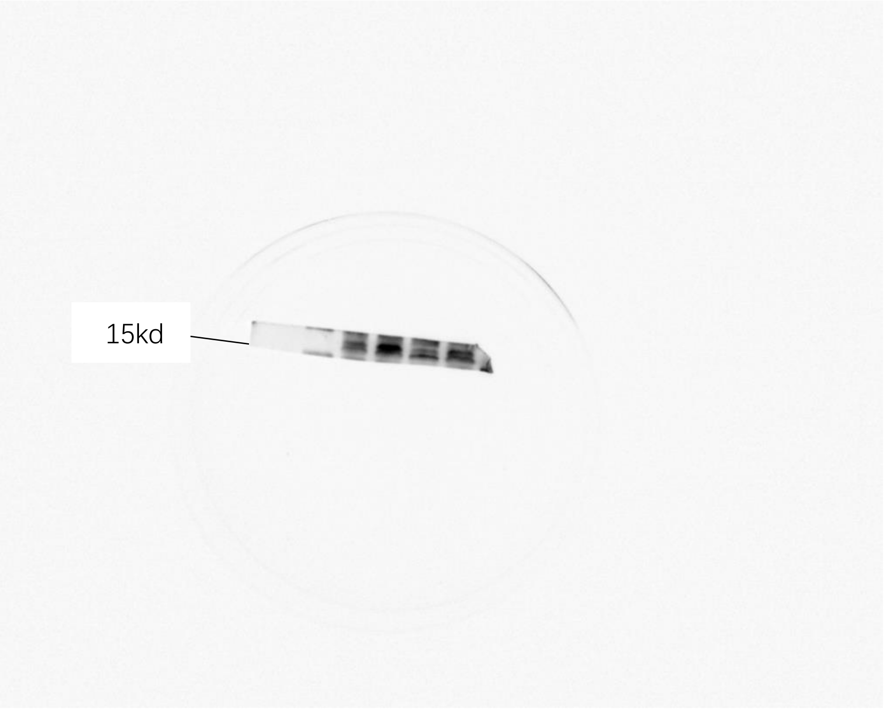

Full unedited blot for Figure5F TNF-α

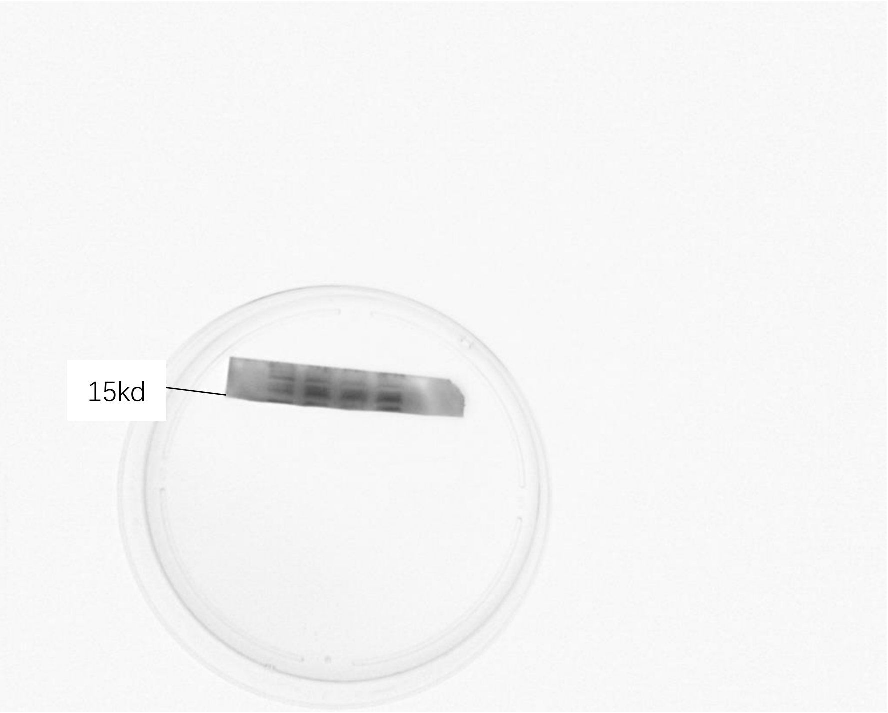

Supplement: Supplementary file 1 — AppendixS1 [file CNS-29-471-s001.pdf]
